# The impact of primary healthcare in reducing inequalities in child health outcomes, Bogotá – Colombia: an ecological analysis

**DOI:** 10.1186/1475-9276-11-66

**Published:** 2012-11-13

**Authors:** Paola A Mosquera, Jinneth Hernández, Román Vega, Jorge Martínez, Ronald Labonte, David Sanders, Miguel San Sebastián

**Affiliations:** 1 Department of Public Health and Clinical Medicine, Epidemiology and Global Health, Umeå University, Umeå, 901 87, Sweden; 2 Postgraduate programs in Health Administration and Public Health, Pontificia Universidad Javeriana, Cr. 40 6-23 P.8, Bogota, Colombia; 3 Faculty of Medicine, Institute of Population Health, University of Ottawa, Ottawa, ON, K1N 6N5, Canada; 4 School of Public Health, University of the Western Cape, P Bag X17, Bellville, 7535, South Africa

**Keywords:** Primary health care, Health status disparities, Inequality, Concentration index, Decomposition, Bogotá

## Abstract

**Background:**

Colombia is one of the countries with the widest levels of socioeconomic and health inequalities. Bogotá, its capital, faces serious problems of poverty, social disparities and access to health services. A Primary Health Care (PHC) strategy was implemented in 2004 to improve health care and to address the social determinants of such inequalities. This study aimed to evaluate the contribution of the PHC strategy to reducing inequalities in child health outcomes in Bogotá.

**Methods:**

An ecological analysis with localities as the unit of analysis was carried out. The variable used to capture the socioeconomic status and living standards was the Quality of Life Index (QLI). Concentration curves and concentration indices for four child health outcomes (infant mortality rate (IMR), under-5 mortality rate, prevalence of acute malnutrition in children under-5, and vaccination coverage for diphtheria, pertussis and tetanus) were calculated to measure socioeconomic inequality. Two periods were used to describe possible changes in the magnitude of the inequalities related with the PHC implementation (2003 year before - 2007 year after implementation). The contribution of the PHC intervention was computed by a decomposition analysis carried out on data from 2007.

**Results:**

In both 2003 and 2007, concentration curves and indexes of IMR, under-5 mortality rate and acute malnutrition showed inequalities to the disadvantage of localities with lower QLI. Diphtheria, pertussis and tetanus (DPT) vaccinations were more prevalent among localities with higher QLI in 2003 but were higher in localities with lower QLI in 2007. The variation of the concentration index between 2003 and 2007 indicated reductions in inequality for all of the indicators in the period after the PHC implementation. In 2007, PHC was associated with a reduction in the effect of the inequality that affected disadvantaged localities in under-5 mortality (24%), IMR (19%) and acute malnutrition (7%). PHC also contributed approximately 20% to inequality in DPT coverage, favoring the poorer localities.

**Conclusion:**

The PHC strategy developed in Bogotá appears to be contributing to reductions of the inequality associated with socioeconomic and living conditions in child health outcomes.

## Background

Although there have been substantial improvements in the average level of morbidity and mortality rates in many countries, health inequalities between and within countries, regions and social groups have widened over the past two decades [1-3]. As a result of this increase, reduction of inequalities is a growing concern for national governments, international organizations and civil society organizations.

In this regard, the World Health Organization has stated that primary health care (PHC) is an effective way for the health sector to contribute to the reduction of social and health inequalities. Its contribution is through the implementation of interventions to address the social determinants (those economic and social conditions - including the structure of the health system- shaped by the distribution of money, power and resources) that influence individual and group differences in health status [4,5]. The best available evidence shows that PHC values and principles –health equity, people-centered care and a central role for communities in health action– can respond to the expectations and challenges of modern societies. Based on these principles, PHC searches for strategies to achieve a more equitable and comprehensive health system and better population health [4,5]. Likewise, the Commission on Social Determinants of Health has suggested that health care services with universal coverage and a focus on PHC could help to generate locally appropriate interventions across the range of social determinants by promoting community participation and intersectoral actions, leading to a reduction in the social causes of health inequalities [6].

Despite Colombia having one of the highest levels of economic and social development in Latin America, it is also one of the countries with the widest socioeconomic and health inequalities [7-10]. The current General System of Social Security in Health (GSSSH) has proposed efficiency, quality and equity as its main objectives. This health system is based on an insurance market with different public-private fund managers and service providers, different regimes of affiliation and several packages of health benefits [11]. The market approach, the public-private combinations of providers and insurers and the regulated competition scheme of the system have created segmentation and fragmentation of health care and barriers to access, particularly for vulnerable groups. These problems are adversely affecting the attainment of the equity goal [12,13].

Bogotá, the capital of Colombia, is the city with the highest degree of economic, political, social and cultural development in Colombia and has one of the largest health care networks in the country. However, in the late 90s and early 2000s, Bogotá faced serious problems of poverty, social disparities and access to health care services that resulted in an increase in health inequalities [13-15]. To face up to this challenge, Bogotá’s government took the decision to formulate and implement public policies and social strategies to address the social determinants of such inequalities. A wide range of programs and interventions, such as economic and nutritional subsidies, housing subsidies, employment generation, building of new nurseries and schools in poor areas and the allocation of school places prioritizing disadvantaged children and young people, were put in place. The health sector, in particular, decided to improve the model of health care, ensuring better access, use and comprehensiveness of the services [15].

One of the strategies included in the district public health policy in 2004 was the implementation of a comprehensive PHC program [15-18]. The strategy was aimed at reinstating the Alma-Ata principles that had been undermined by the Act 100, which reformed the previous national health system into the current GSSSH [14,16,17].

The core of the strategy, from the operational point of view, was the Home Health program (“*Salud a su Casa*” in Spanish). The program works in the network of first-level facilities and public hospitals operating under the authority of the Bogotá District Health Secretariat (DHS). It includes multidisciplinary basic health care teams, comprising a physician, a nurse, two community health workers and an environmental technician. Twelve hundred families are assigned to each team in a geographically defined catchment area (micro-territories)^a^. The program’s intervention began by prioritizing the most vulnerable people, classified as belonging to social strata^b^ 1 and 2, with the aim of gradual expansion to other strata.

Basic health care teams either provide intra or extramural services; their work begins with the application of a household survey for the characterization^c^ of individuals, families and environmental health conditions in order to identify and to prioritize population needs. The program seeks to stimulate the demand for primary health care services, to facilitate access to social and health services and to design specific action plans to provide responses according to the situation of individuals and the community [15-18].

There is evidence that has shown the positive impact of PHC interventions on improving health outcomes and reducing disparities in health. Previous literature reviews have found that PHC is significantly correlated with decreases in mortality rates and increases in life expectancy, and is also related to reducing health disparities measured by income level, geographic location and race/ethnicity [19,20].

In Colombia, several studies have examined the distribution of disparities in health outcomes, and access to and the use of services after the reforms that created the current GSSSH [12,13,21-23]; however, few studies have analyzed the impact of PHC interventions on reducing health inequalities. A descriptive analysis of the improvement in equity of access to and the use of health services carried out by the DHS and the National University of Colombia found positive changes in access to and the use of preventive programs in the micro-territories where the Home Health program worked. However, this analysis reported that there were still inequities in access to outpatient services and preventive care affecting the non-insured population [24]. Another study examining the effect of the PHC strategy on reducing inequalities in health outcomes showed that disparities in the infant mortality rate, the post-neonatal mortality rate and under-5 mortality rate between localities with the best and worst per capita income tended to decrease in the high-coverage group of the Home Health program compared with the low-coverage group [25].

Taking into account that child health outcomes are considered sensitive indicators to measure the results of the PHC interventions [26], and given that monitoring the improvements in children’s health is one of the priorities within the Colombian health policy, we decided to analyze the impact of PHC intervention in reducing inequalities using four child health indicators from which information was available.

This study aimed to evaluate the contribution of the PHC strategy, through the intervention of the Home Health program, in reducing health inequalities in child health outcomes in Bogotá.

## Methods

### Study setting

Bogotá, the capital of Colombia, has 7.035.155 inhabitants and is divided geographically and administratively into 20 localities. According to the district social stratification, 51.2% of the population is classified in strata 1 and 2. By 2010, the Home Health program had achieved 40.36% coverage (1,497,750 people) of the population in strata 1 and 2 through the establishment of 358 basic health care (Home Health) teams.

### Units of analysis and variables

Sixteen of the twenty localities were included in this study as units of analysis. Four localities were excluded because three did not have populations in strata 1 and 2, and the other lacked the socioeconomic information necessary for the analysis.

The variable used to capture the socioeconomic status and living standards was the Quality of Life Index (QLI). The QLI combined 12 variables of access to physical assets organized into four categories:

1. Education and human capital: education of the household head, average education of members aged 12 years or more; young people aged 12–18 years who attended secondary school or university; children aged 5–11 years who attended primary school;

2. Housing quality: material of walls and floors;

3. Access and quality of services: access to health care, water supply and sanitation, kitchen equipment, refuse collection;

4. Household size and composition: number of children under 6 years of age and number of people per room.

Index data were taken from the District Quality of Life survey 2003 and 2007 in publicly available sources of the National Administrative Statistics Department.

The selected child health outcomes included those identified in the literature as sensitive to monitoring PHC implementation and health inequalities [26] and for which information was available. The following indicators were used: infant mortality rate (IMR); under-5 mortality rate; prevalence of acute malnutrition in children under 5 years of age; and vaccination coverage for diphtheria, pertussis and tetanus (DPT) in children under 1 year old. The data were collected from the National Vital Statistics System, the Feeding and Nutrition Epidemiological Surveillance Systems and the Rapid Immunization Coverage Monitoring Registry at the DHS. Data were used with the authorization of the public health department at the District Health Secretariat.

Variables considered as social determinants of health were the Primary Health Care Index (PHCI); the percentage of sewerage coverage and the percentage insurance coverage of the health system. Data to construct the PHCI were provided for the public health department at the DHS, while percentages of insurance and sewerage coverage were taken from the District Quality of Life survey of 2007 from publicly available sources.

The PHCI was constructed using principal component analysis (PCA). This index combined the following variables: Coverage of the Home Health Program; Physician ratio per population; Nurse ratio per population; Community health worker ratio per population. To calculate the PHCI scores by PCA, we used a data set that comprised observations of the variables mentioned earlier during the period 2004-2010. Scores of the PHCI for each year were standardized (transformed linearly giving values from 0 to 100) to allow a more precise classification of the localities into groups. Thus, according to PHCI overall behavior, localities were classified into two groups: the first composed of those localities where the PHCI declined or became stagnant over time (low coverage/group 1) (n = 10); and the second comprised localities that showed a consistent increase of PHCI over time (high coverage/group 2) (n = 6).

The variables mentioned above were considered as social determinants of health because they inform about the socioeconomic conditions under which people live and the situation regarding access to and the use of health care. The assumption is that the inequitable distribution of these variables could be responsible for determining health inequalities. Other socioeconomic variables recognized as social determinants of health outcomes (e.g. income level, availability and access to health services, coverage of social programs aimed at reducing poverty, coverage of economic and nutritional subsidies, mother’s age, mother’s educational level, birth intervals, number of antenatal visits, low birth weight, infant morbidity and duration of breastfeeding, among others) were not included in this analysis because of the lack of information available at the local level.

### Inequality measurement

This part of the analysis aims to describe possible changes in the magnitude of socioeconomic inequalities in child health outcomes after the implementation of the PHC strategy. The analysis was performed using data from 2003 (the year prior to the Home Health program implementation) and 2007 (third year after the implementation); other years were not included in the analysis because of the lack of socioeconomic information.

Concentration curves and concentration indices were used as measures of socioeconomic inequality for the selected child health outcomes. The cumulative percentage of the health variable (child health outcomes – y axis) was plotted against the cumulative percentage of the population, ranked by the socioeconomic variable (QLI – x axis), beginning with the locality with the lowest QLI and ending with the locality with the highest QLI. For each child health outcome, two curves corresponding to the years of the analysis were displayed. For the interpretation of the concentration curves, the curves of each year were compared with the line of 45 degrees (line of equality).

The concentration index (CI), which is directly related to the concentration curve, was calculated to measure and compare the degree of socioeconomic-related inequality in child health outcomes between the periods considered in this analysis. The concentration index is defined as twice the area between the concentration curve and the line of equality (the 45-degree line) and assumes values between -1 and +1. A negative value when the curve lies above the line of equality or a positive value when it lies below the line of equality means that the outcome variable (deaths, malnutrition or vaccination in our case) is concentrated among disadvantaged people (localities with low QLI), while the opposite implies that the variable is concentrated among advantaged people (localities with high QLI). The CI is zero when there is no socioeconomic-related inequality.

The concentration index from grouped data was computed using the formula proposed by Fuller and Lury [27]:

(1) $$\begin{array}{l} \text{C}=({\text{P}}_{1}{\;\text{L}}_{2}-{\text{P}}_{2}{\;\text{L}}_{1}+\left({\text{P}}_{2}{\;\text{L}}_{3}-{\text{P}}_{3}{\;\text{L}}_{2}\right)+\ldots \\ \;+\left({\text{P}}_{\text{T}-1}{\;\text{L}}_{\text{T}}-P_{T}{\;\text{L}}_{\text{T}-1}\right) \end{array}$$

where P_t_ is the cumulative percentage of the sample ranked by socioeconomic status of the localities (group t), and L_t_ is the corresponding concentration curve ordinate.

### Decomposition analysis

The purpose of this analysis was to assess the current contribution of the PHC intervention; for this reason, decomposition analysis was carried out only on data from 2007. The method proposed by Wagstaff et al. [28] was used to decompose the socioeconomic inequality in child health outcomes into its determinants and to estimate how determinants proportionally contribute to the measured inequality. Decomposition of the CI is based on regression analysis of the relationship between the health variable of interest and its correlates. In this study we consider the contribution of the PHC strategy, in addition to some social determinants of the child health outcomes.

According to Wagstaff et al. for any linear additive regression model of health (*y*), such as:

(2) $$\text{Y}=\alpha +\sum_{\text{k}}\;\beta_{\text{k}}{\overset{―}{\text{X}}}_{\text{k}}+\varepsilon$$

the concentration index for *y*, *C*, can be written:

(3) $$\text{C}=\sum_{\text{k}}\;\left(\beta_{\text{k}}{\overset{―}{\text{X}}}_{\text{k}}/\mu \right){\;\text{C}}_{\text{k}}+{\text{GC}}_{\varepsilon }/\mu$$

where μ is the mean of y (health outcome variable), ${\overset{―}{\text{X}}}_{\text{k}}$ is the mean of X_k_ (explanatory variable), C_k_ is the concentration index for X_k_ (defined analogously to C), and GCε is the generalized concentration index for the error term (ε). C is equal to a weighted sum of the concentration indices of the k regressors, where the weight for X_k_ is the elasticity of y with respect to X_k_. The residual component – captured by the last term – reflects the socioeconomic-related inequality in health that is not explained by systematic variation in the regressors across socioeconomic groups.

To decompose the concentration index of child health outcomes and to obtain all of the values required in the equation (3), the following steps were performed:

1. A first step was to estimate a regression model of the health variable to obtain the coefficients of the explanatory variables (B_k_). A logistic regression model for grouped data was used. This type of model is appropriate when the health outcome is binary, as in the case of deaths where the probability is not necessarily linear but linear in the natural logarithm of the odds of death [29,30]. Also, for those cases where the outcome does not have a linear behavior, it is important to note that the attention is focused on the first term of the decomposition equation

(4) $$\text{C}=\sum_{\text{k}}\left(\beta_{\text{k}}\;{\overset{―}{\text{X}}}_{\text{k}}/\mu \right){\;\text{C}}_{\text{k}}$$

2. The next step consisted of calculating the weighted average of the health variable and each of the determinants (μ and ${\overset{―}{\text{X}}}_{\text{k}}$).

3. Then, the concentration indices for the determinants were computed using equation (1) for grouped data.

4. The penultimate step was the computation of the elasticity of the health variable (y) with respect to the determinants (*xk)* by replacing the values obtained in steps one and two in the brackets expression.

(5) $$n_{k}=\left({\text{β}}_{\text{k}}\;\left({\overset{―}{\text{X}}}_{\text{k}}\text{/μ}\right)\right)$$

5. The last step was to quantify the pure contribution of each determinant included in the model in relation to the inequality in the health variable. This absolute contribution of each determinant was computed by multiplying the elasticity related to each determinant and their CIs. The relative (percentage) contribution of each determinant was then obtained by dividing its absolute contribution by the CI of the health outcome.

In decomposition analysis, contributions must be interpreted in relation to the overall CI of the selected health outcome and the CIs of the variables used as determinants. When the overall health CI is negative, a negative absolute contribution indicates a supportive effect of the socioeconomic-related inequality (the determinant analyzed is partly responsible for the inequality). A positive absolute contribution points towards an inequality reducing effect (the determinant analyzed is partly responsible for a reduction in the effect of the inequality). An opposite interpretation is required when the overall health CI is positive.

### Ethics approval

This study was approved by the Ethics Committee of the Department of Postgraduate Programs in health administration and public health, Pontificia Universidad Javeriana, Bogotá.

## Results

### Concentration curves

**Figure 1:**
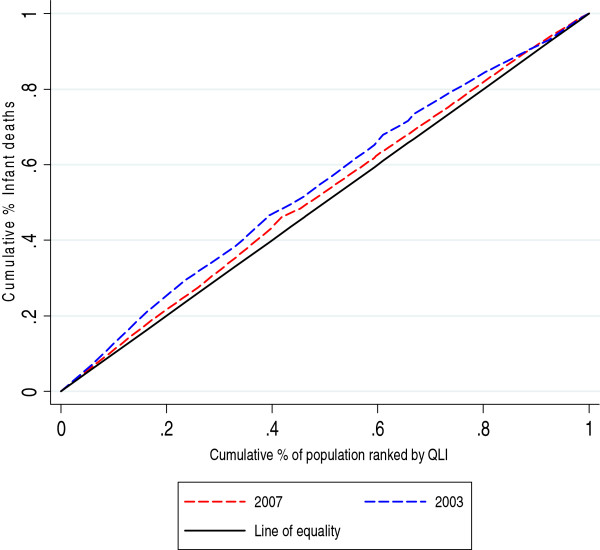
Concentration curve IMR 2003-2007.

**Figure 2:**
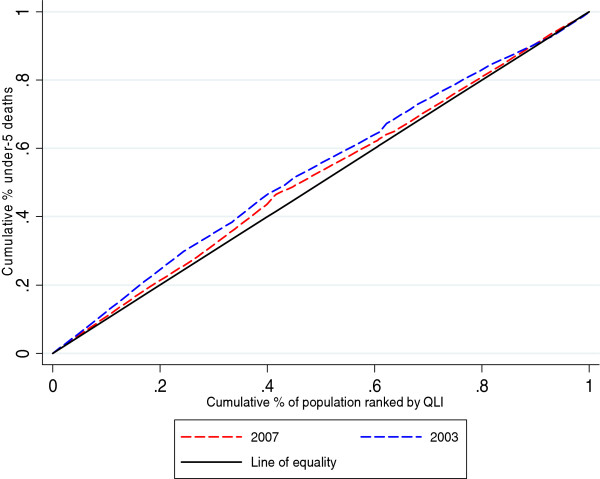
Concentration curve under-5 mortality rate 2003-2007.

Figures 1 and 2 show how the concentration curves lie above the main diagonal, indicating that localities with lower QLI have a greater proportion of infant and under-5 deaths than those with higher QLI. Comparing the two curves (2003-2007), a decrease in inequality was observed for the year 2007.

**Figure 3:**
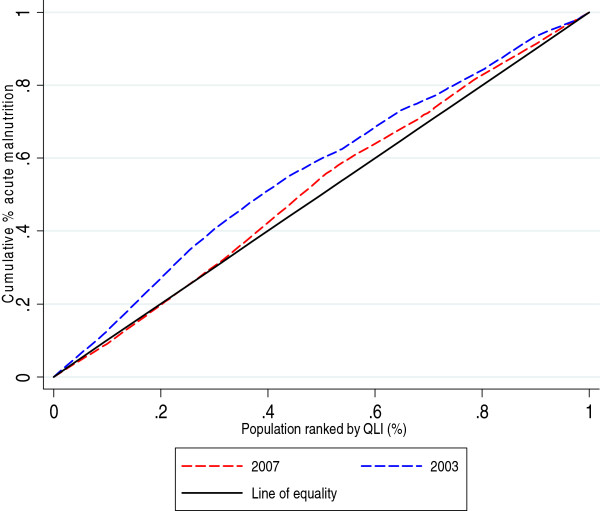
Concentration curve acute malnutrition 2003-2007.

**Figure 4:**
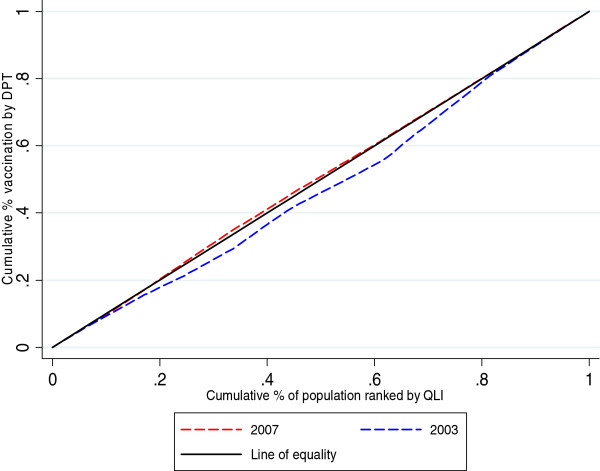
Concentration curve DPT vaccination 2003-2007.

Figure 3 shows that the level of acute malnutrition accumulated faster amongst the localities with lower living conditions than amongst the better-off in 2003. This inequality was reduced in 2007 when the curve shifted closer to the line of equality.

(In Figure 4, the concentration curve of 2003 lies below the main diagonal, indicating that localities with better living conditions (high QLI) have a greater DPT vaccination coverage than those with lower QLI. In 2007, the inequality is reduced and the curve approaches the diagonal line of 45 degrees which indicates a situation of complete equality.

### Concentration index

Table 1 presents the CIs of child health outcomes for 2003 and 2007. CIs of IMR, under-5 mortality rate and acute malnutrition have negative values in both periods, indicating inequalities affecting the localities with lower QLI. The variation in the concentration index observed between the two periods is positive, showing a reduction in inequality in the 2007 period after PHC implementation.

**Table 1:** Concentration indices of child health outcomes 2003-2007

**Variables**	**CI 2003**	**Standard error**	**CI 2007**	**Standard error**	**Change 2007-2003**
Under-5 mortality rate	−0.073*	0.014	−0.032*	0.014	0.041
Infant mortality rate	−0.087*	0.014	−0.038*	0.015	0.049
Acute malnutrition children under -5	−0.128*	0.005	−0.034*	0.007	0.094
Vaccination coverage for DPT ±	0.050*	0.004	−0.007*	0.003	−0.057

* Statistically significant (p < 0.005).

± Signs of values and interpretation of the CI are opposite because this is a favorable health condition.

(This principle of interpretation is used elsewhere in accordance with the original methodology [28]).

The CI for DPT vaccination in 2003 is positive, indicating inequalities that are to the advantage of localities with higher QLI. In 2007, the CI becomes negative but closer to zero, suggesting a more equitable distribution for this indicator.

### Decomposition analysis

Table 2 presents the results of the decomposition analysis for 2007. The column under the heading “concentration index” presents the degree of socioeconomic-related inequality in each of the variables included as determinants. CIs indicate that PHCI is more prevalent among the low QLI localities (CI = -0.024) while insurance and sewerage coverage are more widespread among the localities with higher QLI (0.102 and 0.003 respectively).

**Table 2:** Decomposition analysis of CI of child health outcomes 2007

**Variables**	**Coefficients**	**Elasticities**	**Concentration indices**	**Contributions to C**	
				**Absolute**	**Relative (%)**
**Under-5 mortality rate**					
PHCI	−0.144	−0.330	−0.024	0.008	−24.52
insurance coverage	−0.008	−0.019	0.102	−0.002	6.25
sewerage coverage	0.016	0.054	0.003	0.000	−0.47
Residual				−0.030	94.22
Total				−0.032	
**Infant mortality rate**					
PHCI	−0.061	−0.310	−0.024	0.007	−19.08
insurance coverage	−0.007	−0.038	0.102	−0.004	10.06
sewerage coverage	0.006	0.043	0.003	0.000	−0.31
Residual				−0.035	90.25
Total				−0.038	
**Acute malnutrition in children under 5 years**					
PHCI	−0.008	−0.106	−0.024	0.002	−7.28
insurance coverage	−0.009	−0.124	0.102	−0.013	37.05
sewerage coverage	0.115	2.188	0.003	0.006	−17.58
Residual				−0.030	87.81
Total				−0.034	
**Vaccination coverage for DPT**					
PHCI	0.081	0.063	−0.024	−0.001	20.90
insurance coverage	−0.012	−0.009	0.102	−0.001	13.48
sewerage coverage	0.131	0.147	0.003	0.000	−5.68
Residual				−0.005	71.30
Total				−0.007	

The columns under the heading “contributions to C” present both absolute and relative contributions of each determinant. The PHCI makes absolute contributions in the opposite direction to the overall concentration index of under-5 mortality (0.008), IMR (0.007) and acute malnutrition (0.002), indicating a reduction in the effect of the inequality that affected the disadvantaged localities. The percentages related to these contributions are -24%, -19% and -7% for infant and child mortality and acute malnutrition, respectively.

The interpretation of PHC contributions to vaccination coverage is slightly different. In this case, the overall CI of vaccinations in 2007 had a negative value (CI-0.007), indicating that the distribution favored disadvantaged localities. The negative value of the PHC contribution (-0.001) to the negative overall CI indicates a 20% effect supporting the inequality which favored the poorer localities.

A small part of the inequality in child health outcomes to the advantage of the better-off localities is explained by inequalities in insurance coverage (ranging from 6% in under-5 mortality to 37% in acute malnutrition). Sewerage coverage has a fairly small supportive effect of the equity in under-5 mortality (-0.47%) and IMR (-0.31%) and a larger effect on acute malnutrition (-17.58%).

It is important to note that just a small part of the inequality in child health outcomes to the advantage of the better-off segment of the population is explained by determinants observed in this study. Most of the inequality remains explained by the residual component (ranging from 71% in vaccination for DPT to 94.22% in under-5 mortality). These residuals show the portion of the inequality in health outcomes that cannot be explained by systematic variation in the selected variables of our model across socioeconomic localities. In other words, there are other variables or factors that account for this unexplained part of the inequality as well as other factors that could be part of the explanation of the reductions in the effect of the inequality, but the data for those variables were not included due to the unavailability of the information at the locality level in the period observed.

## Discussion

To our knowledge, this is the first study in Colombia and Latin America that has addressed the question about the contribution of the PHC strategy to reducing health inequalities using both the concentration index and decomposition analysis. Changes in the values of the concentration indices indicates that child health inequalities were reduced in 2007, the period after the implementation of the Home Health program in Bogotá. Results of decomposition analysis allowed us to establish the contribution of the PHC in the reduction of the inequalities, which range from 7% in acute malnutrition to 24% in IMR. The effect of the PHC on reducing inequality remained significant even in the presence of the other examined variables. Results of both the concentration indices and the decomposition analysis suggest that the increase of the Home Health coverage (through the expansion of the number of health personnel per population and interventions of the Home Health program in low-QLI localities) might have a positive effect on reducing disparities in the four indicators studied.

Our results are also consistent with evidence from other contexts where a positive impact of PHC has been reported on improving equity, mainly with regard to the reduction of disparities in access and health outcomes when analyzed by socioeconomic status, ethnicity and geographical location [19,20,31-41].

Although the experiences in the Latin American (LA) context vary regarding the implementation of PHC, studies have shown that the strategy can contribute effectively to reducing gaps in access to and the use of health services associated with socioeconomic differences. This could be partly explained because the implementation of PHC in most LA countries usually began by prioritizing the most disadvantaged groups [20]. In addition to reducing disparities in access, PHC has also shown success in reducing disparities in child deaths. For example, research from Mexico [31] found that certain characteristics of the primary care delivery services (coordination, longitudinality and comprehensiveness) had an important effect on reducing the probability of infant death in socially disadvantaged areas. A study from Bolivia [32] found that a PHC approach focused on solving community needs and the promotion of social participation in socially disadvantaged areas reduced under-5 mortality rates more than in adjacent areas as well as the entire country. Another analysis of nine LA countries, which analyzed the effects of the economic crisis on the trends in infant mortality rates, suggested that those countries where IMR had declined and inequalities had not increased (Chile, Cuba and Costa Rica) were those where access to primary care services had also increased [33]. Specific analyses of the Costa Rican health system reforms have confirmed the results mentioned above. In the case of Costa Rica, the interventions included the expansion of the number of primary care facilities and the creation of basic health care teams assigned to a number of families. These interventions have been associated with increases in life expectancy, decreases in infant mortality rates and a reduction in inequalities in access [34]. For its part, Brazil has provided some evidence suggesting that the “Family Health Program” expansion in the north and north-east regions of the country may have contributed to reducing inter-regional inequalities in infant mortality [35].

Also, comparative country studies have shown the great potential of primary health care to reduce disparities in health outcomes associated with socioeconomic status. Thus, an analysis found that 90% of child deaths were concentrated in 42 countries, and 63% of these deaths could have been prevented by PHC interventions such as comprehensive care for diarrhea, pneumonia, measles, malaria, HIV/AIDS, preterm delivery, neonatal tetanus and neonatal sepsis [19,36]. Other examples of improvements in health equity in developed countries have shown how PHC services have been associated with reductions in socioeconomic inequalities related to ethnicity and geographical location in health outcomes (child mortality and all-cause mortality) and self-perceived health status [37-41]. A literature review, including some studies from the United States of America, highlighted that better primary care development (measured by the number of primary care physicians assigned to the population) is associated with relatively greater effects on health status in socially disadvantaged areas (measured by high levels of income inequality) [19]. This review concluded that areas with high income inequality where PHC was better developed had lower infant mortality rates and better levels of self-perceived health status than areas of high income inequality with less PHC development. In addition, the adverse impact of income inequality on all-cause mortality was significantly reduced by strengthening primary health care interventions [19].

It is important to mention that, although our findings are consistent with other investigations and that the effect of PHC on equity was positive, the magnitude of its contribution to reducing disparities was relatively small for all indicators, especially with regard to acute malnutrition. A possible explanation for the small contribution might be that the indicators analyzed were mostly evenly distributed in 2007 (their concentration index had values very close to zero). It could also be because only data for the third year after the implementation of PHC were included in the present analysis, and a longer period of time would have been required to demonstrate a greater effect.

On the other hand, despite the overall sustained economic growth and poverty declines in Bogotá, inequalities in living conditions at locality levels have not changed substantially. That is the case in some localities where the PHC strategy has been better developed and where simultaneously levels of poverty have increased and coverage of health insurance has lowered [13,42,43]. Consequently, the pace of expansion of the PHC strategy might not have been sufficient to offset the increased vulnerability faced in some localities.

In addition to the previous argument, it is important to highlight that a wide range of social interventions that could affect child health outcomes, such as programs that provide economic and nutritional subsidies, have been implemented simultaneously with the PHC strategy. These interventions could make a greater contribution to reducing disparities, especially in acute malnutrition and child deaths, reducing the equity effect attributable to PHC intervention.

Likewise, it is known that the presence of basic health equipment and the increase in the number of professionals (variables included in the PHCI) do not necessarily ensure better access, use and quality of health services [19]. This is particularly relevant when other economic and administrative barriers (e.g. co-payments, fragmentation in the procurement of services, excessive paperwork requirements to access services and delayed care) persist in the Colombian health system [12-14,21,22], preventing the potential of the PHC strategy to impact on equity.

Finally, some other weaknesses that could also reduce the potential of PHC to affect inequalities include the persistent difficulties in linking all health system stakeholders. PHC has not been able to influence an adequate number of insurers and private providers to improve coordination. Community participation is still shaped according to the rules of the institutions and the rationality of the market, and intersectoral action has not been extended or deepened adequately [14,15].

### Study limitations

Our results are subject to the usual cautions of interpretation of cross-sectional results and the limitations of ecological analysis, which do not provide conclusive evidence of causality. The unavailability of information on a disaggregated level lower than localities (e.g. micro-territories, families or individuals) did not permit us to determine with certainty whether the reductions in disparities in the health outcomes were in favor of the vulnerable population reached by the Home Health program in the locality or if those were an average reduction.

Likewise, the few sources of information available to gather data on other variables recognized as determinants of inequalities in the health outcomes analyzed could be the reasons for residual factors contributing the greatest proportion of the inequalities. The complexity and influence of many social determinants on the health outcomes studied merit further analysis [22,26].

Also, the analysis of the PHC contribution to reducing disparities requires further research, including additional variables that allow a better understanding of the effectiveness of its component health interventions (e.g. health education at facilities, home visits by community health workers, reference to social services) to discover which PHC interventions are contributing to improvements in health equity.

On the other hand, the periodicity of the socioeconomic information used in this analysis (which is collected every four years) reduced the possibility of including more observation periods, and this could have limited a better appreciation of the possible effect of PHC in reducing disparities. Indeed, measuring the implementation of the strategy only three years after its implementation might not be sufficient to measure its true impact on equity.

The QLI reflects a particular definition of living standards that is not necessarily equivalent to wealth or income levels, which are the variables frequently used to rank population in this kind of analysis. The QLI also summarizes many variables grouped in different dimensions. Different rankings of localities might be obtained if other indicators, besides QLI, were used.

One recognized limitation of the decomposition analysis is that the method relies on linear models [30]. In our case, the model used was a non-linear one due to the nature of the outcomes analyzed. The limitations related to the use of non-linear models, grouped data and a small number of explanatory variables could result in approximations to partial effects of these determinants on health outcomes.

## Conclusions

The analysis of changes in the distribution of child health outcomes measured by concentration curves and concentration indices showed a reduction in inequality after the implementation of the PHC strategy in Bogotá. The PHC strategy, through the Home Health program, appears to be contributing to reductions in disparities associated with socioeconomic and living conditions in under-5 mortality, IMR, acute malnutrition and vaccination coverage for DPT.

The health policies and the PHC strategy developed in Bogotá seem to be helping to improve health equity (taken here to mean the reduction in preventable health inequalities). This suggests that, even in adverse contexts such as those persisting in the Colombian health system, it is possible that governments committed to the goal of health equity could obtain good results when PHC is prioritized in their agenda.

The policy implication is that the results presented by this analysis are providing timely, relevant information to support health evidence-based policies aimed at promoting changes to transform the fragmented and segmented health system to one based on the PHC approach. Significant efforts to overcoming the financial logic of the Colombian health system as well as the implementation of social policies largely focused on intersectoral work and community participation are imperative.

## Endnotes

^a^Micro-territory is a geographically subdivision created to delineate catchment areas of the home health program within Bogota’s localities. A micro-territory is comprised by twelve hundred families located in a determined area (usually a neighborhood) which are assigned to a basic health care team.

^b^Strata classify socioeconomic groups from 1 to 6, 1 being the lowest and 6 the highest. This classification determines the taxes and prices of utilities (gas, water, electricity) as well as access to health services, among others.

^c^Characterization is the first activity carried out by the basic health care teams in the “Home Health” program in order to include the families of strata 1 and 2. This consists of the application of a survey. which identifies the socioeconomic and health conditions of the family.

## Competing interests

The authors declare that they have no competing interests.

## Authors’ contributions

PM, JH, RV and JM conceived the study, participated in the data collection, analysis, interpretation of the data and drafted the manuscript. RL and DS participated in study design and coordination and helped to draft the manuscript. MSS contributed to the analysis and interpretation of the data and revised the manuscript for clarifications. All authors approved the final draft.
